# Comparative Kinetic Analysis of Closed-Ended and Open-Ended Porous Sensors

**DOI:** 10.1186/s11671-016-1614-3

**Published:** 2016-09-13

**Authors:** Yiliang Zhao, Girija Gaur, Raymond L. Mernaugh, Paul E. Laibinis, Sharon M. Weiss

**Affiliations:** 1 Interdisciplinary Graduate Program in Materials Science, Vanderbilt University, Nashville, TN 37235 USA; 2 Department of Electrical Engineering and Computer Science, Vanderbilt University, Nashville, TN 37235 USA; 3 Department of Biochemistry, Vanderbilt University Medical Center, Nashville, TN 37232 USA; 4 Department of Chemical and Biomolecular Engineering, Vanderbilt University, Nashville, TN 37235 USA

**Keywords:** Flow-through, Membrane, Porous silicon, Microfluidics, Finite element simulation, Adsorption kinetics, Biosensor, Protein adsorption

## Abstract

Efficient mass transport through porous networks is essential for achieving rapid response times in sensing applications utilizing porous materials. In this work, we show that open-ended porous membranes can overcome diffusion challenges experienced by closed-ended porous materials in a microfluidic environment. A theoretical model including both transport and reaction kinetics is employed to study the influence of flow velocity, bulk analyte concentration, analyte diffusivity, and adsorption rate on the performance of open-ended and closed-ended porous sensors integrated with flow cells. The analysis shows that open-ended pores enable analyte flow through the pores and greatly reduce the response time and analyte consumption for detecting large molecules with slow diffusivities compared with closed-ended pores for which analytes largely flow over the pores. Experimental confirmation of the results was carried out with open- and closed-ended porous silicon (PSi) microcavities fabricated in flow-through and flow-over sensor configurations, respectively. The adsorption behavior of small analytes onto the inner surfaces of closed-ended and open-ended PSi membrane microcavities was similar. However, for large analytes, PSi membranes in a flow-through scheme showed significant improvement in response times due to more efficient convective transport of analytes. The experimental results and theoretical analysis provide quantitative estimates of the benefits offered by open-ended porous membranes for different analyte systems.

## Background

In recent years, porous materials have attracted a great deal of interest in research fields such as energy conversion [1, 2], drug delivery [3, 4], and medical diagnostics [5, 6] due to their large internal surface area and tunable pore size distributions. Open-ended pores present in porous membranes are widely used in micro-fuel cells as gas diffusion layers and proton exchange membranes [7, 8], and many studies have been carried out to investigate the mass transport properties of porous membranes in fuel cell applications [9, 10]. Similarly, the out-diffusion of drugs from porous particles has been investigated [11, 12]. However, in biosensing applications, the use of open-ended porous membranes is not common and has not been widely studied. Most biosensing approaches that incorporate microfluidic systems utilize closed-ended pores due to an ease of fabrication and thus rely only on diffusive transport of analytes in solution to the inner pore sensing surfaces [13–15]. In this flow-over configuration, the diffusive flux into each individual pore can be as slow as a few molecules per second for molecules whose size approaches that of the pore opening. In this configuration, the majority of the molecules of interest are swept through the channel and past the sensor without interacting with the inner pore sensing surfaces [16, 17].

Open-ended porous membranes [18–20] and nanohole arrays [21–23] offer the possibility to overcome inefficient mass transport and achieve fast sensor response by allowing analytes to flow through the pores and interact more favorably with their inner surfaces. Nanoporous membranes have been prepared in materials such as silicon [24–26], alumina [20, 27, 28], titania [29, 30], and various polymers [31, 32]. An enhancement in the rate of mass transport through such membranes has been reported for porous alumina with the use of fluorescently labeled species [20]. An emerging interest in lab-on-chip biosensing technologies has been focused on label-free refractometric-based sensors in order to avoid the additional processing and cost associated with the use of fluorescent markers [33, 34]. Among the various porous materials, porous silicon (PSi) has been considered as a favorable material for constructing low-cost label-free optical biosensors due to the easy manipulation of its pore sizes, optical properties, and surface chemistries [35–37]. PSi membranes have been previously used to separate molecules [25, 38], construct fuel cells [39, 40], and investigate transmembrane proteins [41]. Open-ended PSi membranes have been fabricated by methods including anodization to etch thinned areas of silicon wafers (“etch-through” approach) [39–41] and electropolishing to separate an anodized PSi film from the substrate (“lift-off” approach) [19, 26, 42]. Challenges in these fabrication approaches arise for the formation of robust and high-quality multilayer optical structures. For example, the “etch-through” approach leads to a porosity gradient caused by carrier depletion as the etching proceeds, which affects the optical properties of the structure, while the “lift-off” approach is hindered by low repeatability and fragility of the free-standing membrane. As a result, most PSi membranes reported so far have served solely as flow-through nanochannels and have not incorporated any multilayered structures.

In order to realize a label-free flow-through sensing approach with PSi, we developed an open-ended, multilayered, optical microcavity structure fabricated by standard silicon processing that is compatible with integration in on-chip sensor arrays [43]. Our experimental results demonstrated proof-of-concept flow-through biosensing and a sixfold improvement in sensor response time compared to the conventional closed-ended, flow-over PSi sensor when monitoring the streptavidin-biotin binding process [43]. Despite these promising results, implementing flow-through sensing requires many important design considerations and it is necessary to determine the kinetic conditions under which an open-ended, flow-through porous membrane offers advantages over simpler, closed-ended, flow-over porous sensors. In this work, the benefits of using PSi microcavity membranes for flow-through sensing as compared to closed-ended PSi microcavity films in the flow-over scheme are quantified by evaluating the relevant transport and reaction influences in open-ended and closed-ended pores using finite element simulations. The effects of flow velocity, bulk analyte concentration, analyte diffusivity, and adsorption kinetics on sensor response are simulated and compared between the two different flow schemes. An experimental demonstration of flow-over PSi microcavity sensors vs. flow-through PSi microcavity membrane sensors upon exposure to analytes with different sizes is also presented to validate a subset of the calculated results. We note that the computational analysis is not limited to PSi, but can be more broadly applied to evaluate the analyte transport and time response of other material systems comprised of nanopores.

## Methods

### Theoretical Model and Numerical Simulation

The transport and adsorption kinetics of both the flow-over (i.e., closed-ended pores) and flow-through (i.e., open-ended pores) schemes were simulated using the finite element method software COMSOL Multiphysics (v 4.2) under the assumption of steady state 2D laminar flow. The simulation, based on standard COMSOL modules used in other studies [13, 16, 44, 45], involves calculating the velocity profile and the concentration distribution of analyte solution in the micro-channel by solving the Navier-Stokes and convection-diffusion equations. The adsorption kinetics of various analyte systems is later combined with mass transport in the porous sensing region to determine the sensor response times of the two different flow schemes.

The following parameters were used in the COMSOL simulations: inlet velocity *u*_0_ = 10^−6^–10^−2^ m/s, reference pressure *p*_ref_ = 1 atm, analyte concentration in bulk flow *c*_0_ = 10^−4^−10^−3^ mol/m^3^, diffusivity *D* = 10^−11^−10^−9^ m^2^/s, adsorption rate constant *k*_*a*_ = 10^2^−10^4^ m^3^/mol s, desorption rate constant *k*_*d*_ = 1 × 10^−6^ s^−1^, concentration of adsorption sites at the sensing surface *b*_0_ = 1 × 10^−7^ mol/m^2^, number of pores = 500, and lateral extent of porous region *w* = 15 μm. The pore geometry used in the simulations was chosen to approximate the experimentally fabricated PSi sensors: pore diameter = 25 nm, pore separation = 5 nm, and pore depth *h* = 4 μm. The flow cell dimensions are height *H* = 60 μm and channel length *L* = 100 μm in the flow-over scheme with closed-ended pores, and *H* = 60 μm and *L* = 60 μm in the flow-through scheme with open-ended pores. Water is considered as the medium inside the flow cell along with the molecules under test. The models are meshed using triangular elements with refined mesh sizes in the porous area; the maximum element size is 10 nm and the minimum element size is 0.9 Å.

### Fabrication of Open- and Closed-Ended PSi Microcavities

**Fig. 1:**
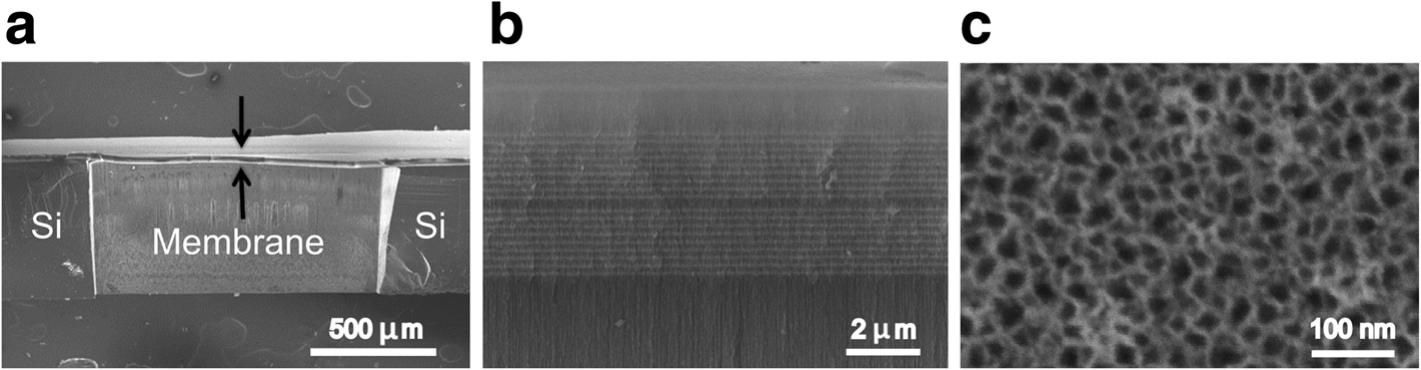
SEM images of a fabricated PSi membrane. **a** Cross-sectional SEM image of the open-ended PSi membrane region and the surrounding silicon substrate. The area below the ~15-μm-thick membrane is open to allow analyte to flow through the porous membrane. **b** Magnified cross-sectional SEM image showing the PSi layers comprising the microcavity and the surrounding sacrificial layers. **c** Top view SEM image of the PSi membrane

The PSi structures employed in this work consist of a multilayer microcavity structure to enable highly sensitive optical measurement. The microcavity multilayer centered at *λ* ≈ 650 nm contains periods of alternating high (H)- and low (L)-porosity layers with a configuration of (L H)^9^(H L)^9^. All layers have an optical thickness equal to *λ*/4, while the central defect layer has an optical thickness of *λ*/2. A wafer-scale silicon etching system with an electrolyte containing 15 % hydrofluoric acid solution was used to form the PSi microcavity on double-side-polished, boron-doped silicon wafers (<100>, 0.01–0.02 Ω cm, 500–550 μm). The anodization current densities were 48 and 20 mA/cm^2^, for H and L, respectively. Sacrificial layers etched at 48 mA/cm^2^ were included at the top and bottom of the microcavity, which were necessary for the open-ended membranes to provide process tolerance during membrane fabrication and mechanical support but were also included in the closed-ended PSi samples for consistency. All PSi wafer samples were oxidized for 5 min at 500 °C in air. Standard lithographic techniques were carried out to realize 1 mm × 1 mm open-ended PSi membranes. Briefly, contact lithography and reactive ion etching (RIE) were used to open windows on the top side of the samples. A second, aligned contact lithography step was carried out to pattern the back side of the samples followed by a deep RIE Bosch etch process to fully open the membranes. Complete fabrication details have been reported in ref. [43]. Scanning electron microscope (SEM) images presented in Fig. 1 were used to estimate the pore diameters and layer thicknesses for the fabricated PSi microcavity structures. The resulting PSi microcavity was approximately 4-μm thick, with a pore diameter ≈30 ± 5 nm in the high-porosity layers and a pore diameter ≈20 ± 5 nm in the low porosity layers. The total thickness of the open-ended PSi membrane including the top and bottom sacrificial layers was approximately 15 μm. The membranes showed good mechanical stability and could easily withstand the pressure in flow-through experiments with flow velocities up to 15 μL/min.

### Microfluidics Integration and Real-Time Adsorption Measurement

Microfluidic channels were attached to the PSi microcavities to facilitate real-time optical monitoring of molecular adsorption activities within the porous matrix. Standard soft lithography techniques were used to fabricate PDMS micro-channels with the dimension of 7 mm × 2 mm × 60 μm following the procedures described previously [45, 46]. A single flow channel was attached after oxygen plasma treatment to the top of the closed-ended PSi microcavities to realize the flow-over scheme. The PSi membrane samples were sealed between two microfluidic channels where the inlet for analyte solution was present in the upper channel and the outlet was in the bottom channel. In this way, injected solution was forced to pass through the open-ended pores to realize the flow-through scheme. A syringe pump was used to generate a constant flow of analyte solution in the micro-channels. A fiber-coupled Ocean Optics USB4000 CCD spectrometer with a 1-mm spot size was used to collect reflectance spectra using an integration time of 10 ms, similar to the reflectance setup reported in ref. [47]. The measured reflectance spectra of PSi microcavities follow a similar shape to our prior results [48].

To evaluate the transport and adsorption kinetics in the closed-ended and open-ended PSi microcavities, 3-aminopropyltriethoxysilane (3-APTES), horseradish peroxidase (HRP), and catalase (CAT) were employed as representative analytes for their different molecular sizes and varied diffusivities in the range of 10^−9^−10^−11^ m^2^/s. 3-APTES is a small aminosilane with a molecular weight of 221 Da and a length ≈0.8 nm. A 2 % 3-APTES solution in DI water and methanol was continuously injected for reaction with the oxidized PSi. HRP is a 44-kDa glycoprotein with a diameter ≈4 nm and isoelectric point of 7.2. A 1 mM sodium acetate buffer at pH 5 was used to prepare a 5-μM HRP solution. At pH values lower than its isoelectronic point, HRP molecules become positively charged; and they can therefore electrostatistically adsorb onto the negatively charged oxidized PSi. CAT is a common enzyme that catalyzes the decomposition of hydrogen peroxide to water and oxygen. It is a relatively large 247.5-kDa molecule with a diameter ≈10.2 nm and isoelectric point of 5.4. PBS buffer at pH 7 (8 g NaCl, 0.2 g KCl, 1.44 g Na_2_HPO_4_, and 0.24 g KH_2_PO_4_ in 1 L of DI water) was used to dilute the CAT solution to a concentration of 5 μM. All solutions were injected at 5 μL/min and a rinsing step with DI water was performed after each molecular adsorption step to remove unbound species.

## Results and Discussion

### Transport and Adsorption Kinetics in Closed-Ended and Open-Ended Porous Sensors

**Fig. 2:**
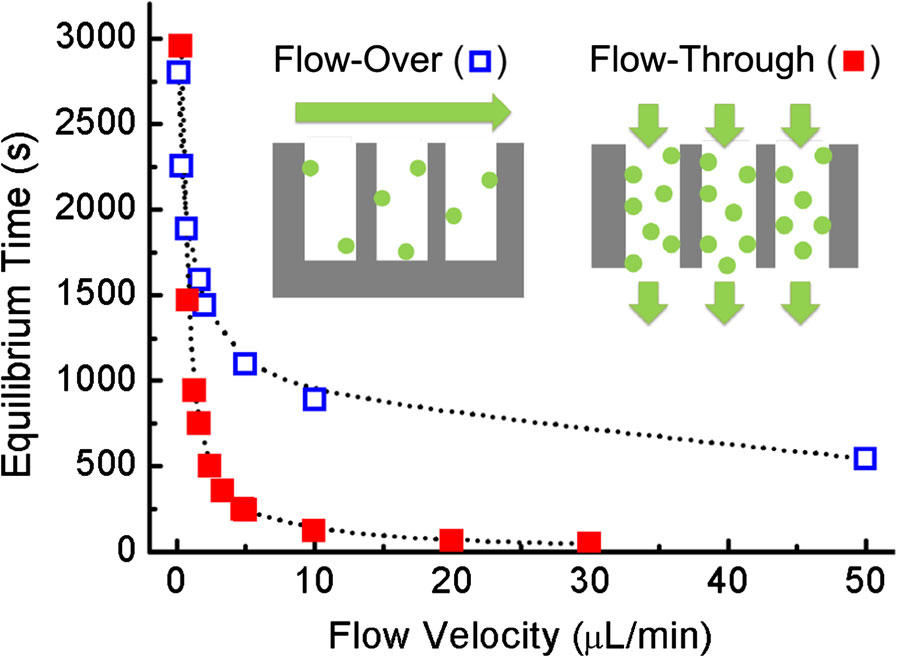
Equilibrium time of both flow schemes as a function of the flow velocity. Analyte concentration = 1 μM. *Inset*: schematic illustrations of closed-ended pores in the flow-over format and of open-ended pores in the flow-through format

Analyte adsorption requires two factors: transport of analyte to the sensor surface and a subsequent adsorption process. In an ideal case, the adsorption kinetics are rapid and mass transport replenishes analytes to the sensor surface sufficiently quickly for adsorption reactions to continue. However, due to the high aspect ratio of the nanopores, porous sensors presenting closed-ended pores that only allow a conventional flow-over scheme for analyte delivery usually operate in a diffusion-limited regime, where the rate that analyte molecules are delivered to the sensing surface is insufficient and leads to slow sensor response times [16, 17]. Porous sensors with open-ended pores can support a flow-through analyte delivery scheme in which solutions pass through the open-ended pores (Fig. 2, inset). Here, analytes are delivered to the sensor surface not only by diffusion but also by convection. The flow-through scheme enhances analyte transport in porous regions as confirmed by the absence of depletion zone and the lack of lateral variations in the simulated concentration distribution, while for the flow-over scheme, most analytes do not reach the porous sensing area due to the formation of the depletion zone [43]. In order to study and compare the efficiency of analyte transport in flow-through porous sensors with open-ended pores and closed-ended porous sensors in the flow-over scheme, numerical simulations under different flow velocities and analyte concentrations were performed.

Figure 2 shows the equilibrium time of closed-ended and open-ended porous sensors in their respective flow schemes as a function of the flow velocity when analyte diffusivity *D* = 1 × 10^−11^ m^2^/s, which is representative of large protein molecules [49, 50], and a bulk analyte concentration is of *c*_0_ = 1 μM. The equilibrium time is defined as the time when all available sites on the sensor surface have been occupied by analytes and the sensor response reaches saturation. The flow velocity is given in dimensional form by multiplying the input velocity specified earlier for the COMSOL simulation by the channel height of 60 μm and width of 2 mm. To focus on the role of transport, rapid adsorption kinetics with *k*_*a*_ = 1 × 10^4^ m^3^ mol^−1^ s^−1^ and *k*_*d*_ = 1 × 10^−6^ s^−1^ were applied so that analytes adsorb to the sensor surface immediately. As indicated in Fig. 2, when there is no flow (e.g., flow velocity = 0 μL/min), both flow schemes represent stagnant analyte solutions, where the replenishment of consumed analyte near the sensor surface relies only on diffusion. Likewise, at low flow velocities (<1 μL/min), both flow schemes operate in a diffusion-limited regime resulting in similarly long equilibrium times. The equilibrium time shortens when flow velocity increases due to enhanced mass transport. However, at flow velocities greater than 10 μL/min, further increases in the flow velocity become less effective for both flow schemes because the sensor response approaches the reaction-limited regime where mass transport supplies analyte quicker than the sensor can adsorb them. At flow velocities between 5 and 10 μL/min in which neither flow scheme is reaction-limited nor diffusion-limited, the equilibrium time is reduced fivefold by employing the flow-through scheme with open-ended pores.

**Fig. 3:**
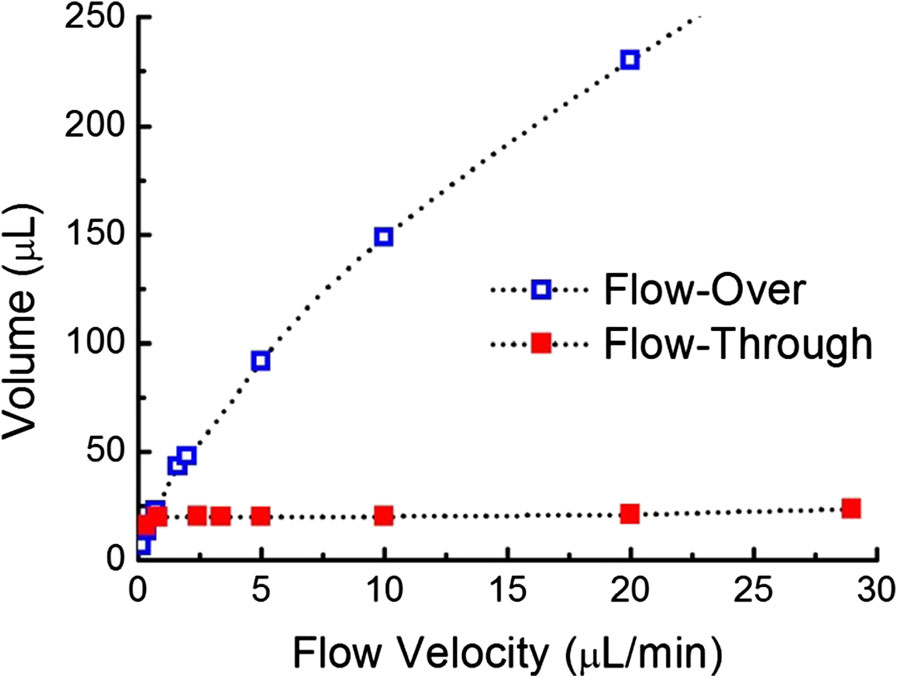
Total volume of analyte solution required in closed-ended, flow-over porous sensors and open-ended, flow-through porous sensors to reach equilibrium at different flow velocities. Analyte concentration = 1 μM

While increasing the flow rate reduces the response time for porous sensors, the amount of analyte consumed must be considered. In biosensing applications, when sample availability is limited, minimizing the total analyte volume consumed is especially important. Figure 3 shows the total volume of analyte solution required for both flow schemes to achieve their equilibrium response for different flow velocities. Analyte diffusivity, concentration, and rate constants were the same in Fig. 3 as in Fig. 2. The volume of analyte consumed was calculated by multiplying the dimensional flow velocity by the equilibrium time. The total required volume of analyte solution in the flow-over scheme with closed-ended pores increases rapidly in response to the increases in flow velocity as most of the analyte molecules flow past the sensor region without entering the pores. However, in the flow-through scheme with open-ended pores, the required volume of analyte changes little with flow velocity. This result can be explained by recalling the assumption of rapid adsorption kinetics used in the simulation and considering that the flow-through configuration forces all analytes to pass through the nanopores in close proximity to the pore walls. Accordingly, the flow-through scheme is particularly favorable for porous sensors as it facilitates rapid response time and a reduced analyte volume.

**Fig. 4:**
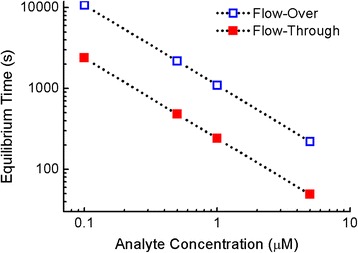
Effect of analyte concentration on equilibrium time. Flow velocity = 5 μL/min. The equilibrium times of flow-over sensors with closed-ended pores and flow-through sensors with open-ended pores show an inverse dependence on analyte concentration

Next, we evaluate how the bulk concentration of analyte in the micro-channel affects analyte transport in both flow schemes. Figure 4 shows the simulated equilibrium time at different target analyte concentrations ranging from 0.1 to 5 μM with a fixed flow velocity of 5 μL/min. The same analyte diffusivity and rate constants were used in Fig. 4 as in Figs. 2 and 3. In both flow schemes, analyte at a lower bulk concentration of 0.1 μM requires approximately 50 times longer to reach equilibrium than analyte at a higher concentration of 5 μM. In agreement with the assumption of first-order Langmuir kinetics [51], at a sufficiently low desorption rate constant, the equilibrium time in both flow schemes is inversely proportional to the analyte concentration, as shown in Fig. 4. We note that although the flow-through configuration with open-ended pores maintains approximately fivefold improvement in equilibrium time throughout the simulated concentration range, the impact on time saving by the flow-through scheme is stronger for analyte at lower bulk concentrations. At a low analyte concentration of 0.1 μM, the equilibrium time is reduced from approximately 3 h to 40 min by replacing closed-ended, flow-over porous sensors with open-ended, flow-through porous sensors. For analytes at a higher concentration of 5 μM, although the same improvement ratio is achieved, the open-ended sensors only reduce the equilibrium time by less than 3 min. Thus, the flow-through sensing approach is especially advantageous for providing more reasonable sensor response times when detecting dilute samples.

**Fig. 5:**
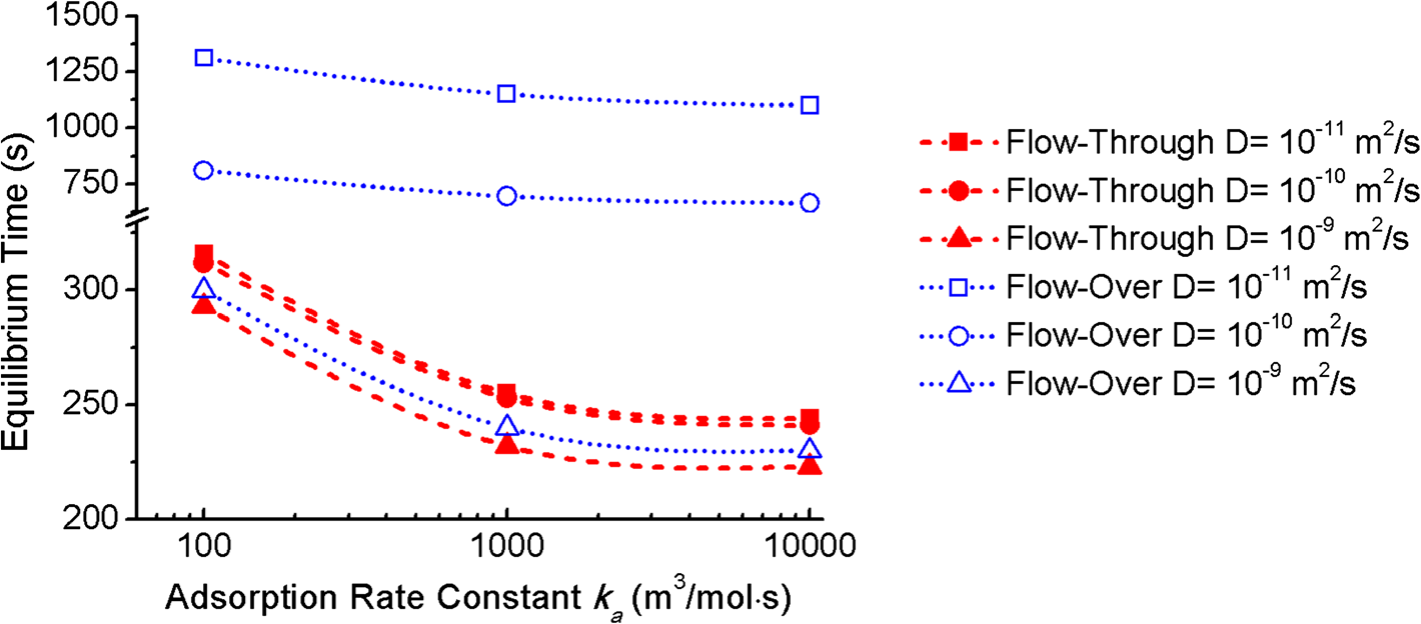
Equilibrium time of both flow schemes for analytes with different diffusivities *D* and adsorption rate constants *k*_*a*_. Flow velocity = 5 μL/min and analyte concentration = 1 μM

Analyte size plays an important role in their transport within porous matrices. Larger molecules have slower diffusivities; for instance, the diffusion constant for the protein bovine serum albumin (69 kDa) in an aqueous solution is approximately 1 × 10^−11^ m^2^/s [49], while those for small ions and for ethanol in water are around 10^−9^ m^2^/s [52]. The transport of large molecules within porous materials is slower than in bulk solution due to hindered diffusion in nanoscale pores. As a result, molecular diffusivities in nanopores depend not only on the nature of the molecules themselves but also on the geometry and morphology of the porous materials [53, 54]. The equilibrium times for porous sensors having closed-ended pores in the flow-over scheme and open-ended pores in the flow-through scheme was investigated for analytes with different diffusivities and adsorption kinetics. Adsorption rate constants of *k*_*a*_ > 10^2^ m^3^/mol s are typical for most molecular adsorption events, we consider adsorption rate constants between 10^2^ and 10^4^ m^3^/mol s in our model. For these conditions, Fig. 5 shows that the equilibrium time is reduced with faster adsorption kinetics and diffusivities. Similar to the impact of porous structures on molecular diffusivities, the effective adsorption rate in nanoscale pores can be 10^2^–10^4^ times smaller than the adsorption rate on flat surfaces due to mass transport limitations [45, 55]. Moreover, adsorptions with large rate constants are more significantly affected in nanoporous regions compared to adsorptions with small rate constants [16], resulting in a reduced dynamic range of effective adsorption rate constants in porous sensing regions. Therefore, although Fig. 5 considers a three order of magnitude difference in adsorption rate constant, this rate constant is the bulk one and the range of effective adsorption rate constants in the nanopores is smaller. Consequently, although the inverse dependence of equilibrium time on adsorption rate constant is clearly shown in Fig. 5, as is expected from first-order Langmuir kinetics [51], the time to reach equilibrium does not show a strong dependence on the adsorption rate constant. Examining the role of diffusivity, we find the benefit of the flow-through scheme with open-ended pores is less significant for small analytes (i.e., molecular weight <1 kDa) with diffusivities on the order of 10^−9^ m^2^/s because their small molecular size provides relatively fast diffusive transport rate in both systems. For analytes with bulk diffusivities around 10^−10^ m^2^/s, the flow-through configuration provides less than threefold improvement in simulated equilibrium times compared to the flow-over scheme. When applied to larger analytes (i.e., molecular weight >100 kDa) with slow diffusivities around 10^−11^ m^2^/s, the flow-through scheme shows an approximately five times faster equilibrium time than the flow-over scheme, which is consistent with the results in Figs. 2 and 4. For those large analytes, the flow-through configuration offers significant benefit of enhancing the mass transport efficiency by providing convective transport of analytes through the open-ended pores. The enhanced convective transport of analytes in the open-ended pores causes the sensor response time to be less dependent on analyte diffusion rate; therefore, large molecules with low diffusivities reach equilibrium almost as rapidly as small molecules in the flow-through configuration.

In the above analysis, the 2D simulation space included 500 straight pores with uniform diameters of 25 nm in order to keep the computational time to manageable levels. Actual porous sensors contain many more pores and often with a complicated morphology. In the adsorption experiments detailed in the following section, the PSi sensors contain approximately 10^9^ pores that alternate in layers of high and low porosity with slightly different average pore diameters. The increased amount of pores and the tortuosity in the PSi matrix impact both the diffusion and adsorption of molecules in the nanoscale pores. Therefore, the simulated results for open-ended and closed-ended porous sensors serve as a guide to estimate the relative trends of the performance for the flow-through and flow-over sensing formats. Because the results have a strong dependence on the geometry and morphology of the porous matrix, the exact results from simulation cannot be compared directly with those obtained in experiments.

### Adsorption Kinetics in Flow-Over and Flow-Through PSi Microcavities

**Fig. 6:**
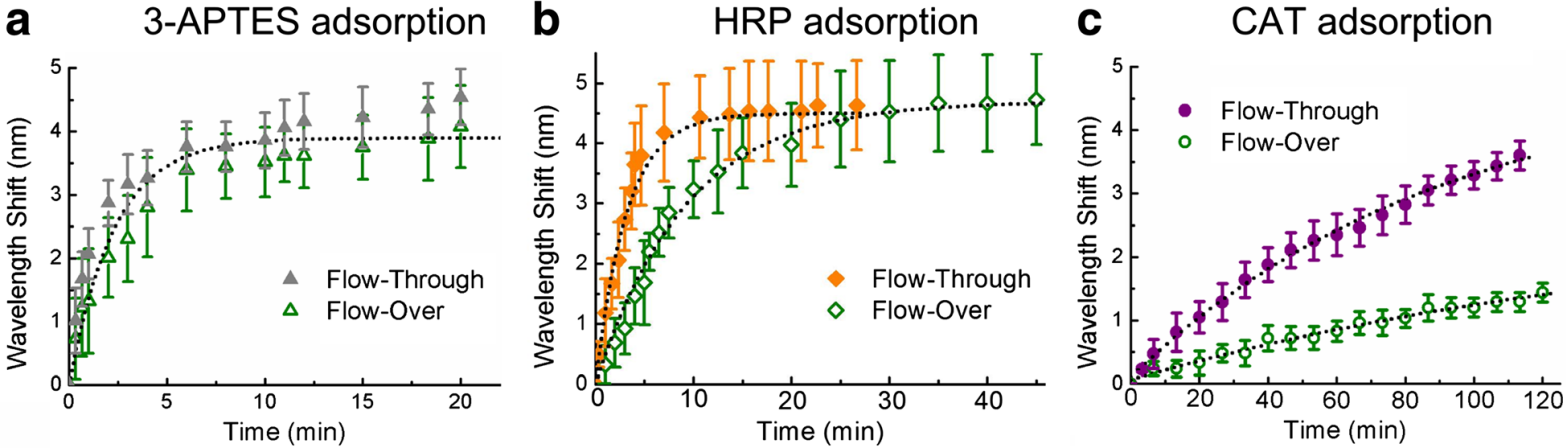
Comparison of real-time PSi microcavity response for closed-ended pores in the flow-over scheme and open-ended pores in the flow-through scheme. Time-dependent PSi microcavity resonance wavelength shifts upon exposure to **a** 3-APTES, **b** HRP, and **c** CAT. *Solid symbols* are experimental data obtained from an open-ended, flow-through PSi microcavity membrane; *hollow symbols* are experimental data from a closed-ended flow-over PSi microcavity. *Dotted lines* provide guides to the eye

In order to validate the results of the finite element simulations, molecule adsorption experiments were carried out on both flow-over and flow-through PSi microcavities. When analytes are captured inside the porous matrix, the effective refractive index of PSi increases, providing a shift of the microcavity resonance to longer wavelengths. In this way, analyte binding or adsorption can be quantitatively determined by monitoring the shift of the reflectance spectra. The microcavity structure enables highly sensitive label-free optical sensing as a result of strong light-matter interaction between localized electric fields in the central cavity region and present molecules [56]. The application of PSi microcavities as biosensors is challenged by an associated long response time due to hindered analyte diffusion in the low porosity layers whose average pore size is 20 nm. For the detection of large molecules with slow diffusive transport, the use of the PSi microcavity as a sensor platform therefore becomes impractical. To illustrate this issue, we first experimentally evaluated the sensing performance of conventional on-substrate PSi microcavities with closed-ended pores to molecules of different sizes in order to estimate for which range of molecule size and diffusivity the microcavity response time becomes prohibitively long (Fig. 6, green hollow symbols). We used a large, 10.2-nm diameter CAT protein, a 4-nm diameter HRP protein, and a small 0.8-nm 3-APTES molecules as model analytes to study the effect of analyte size on sensor response. The sensor response time is the time required to reach an equilibrium state wherein the average surface concentration of analytes immobilized on the sensor does not change as represented by a saturation of the wavelength shift. The attachment of 3-APTES involves a silanization process, while protein adsorption is charge-based. Our simulation results in Fig. 5 suggested that due to mass transport limitations, the response time of the porous sensor is dominated by analyte diffusivity and is only weakly dependent on adsorption rate constants. Therefore, the sensor response for adsorption of 3-APTES and proteins is primarily determined by their different sizes (i.e., diffusivities) rather than adsorption mechanisms. The adsorption of 3-APTES and HRP quickly reached saturation in approximately 10 and 20 min, respectively, while the adsorption of the large CAT protein was slow. This trend corresponds well to the simulation results presented in Fig. 5 that show for larger molecules that diffuse more slowly, the closed-ended PSi sensor takes longer to reach equilibrium. For the CAT protein, approximately 1.5-nm wavelength shift was measured using the closed-ended pore microcavity after 120 min of continuous analyte injection. The slow response of this PSi microcavity to CAT adsorption is attributed to the corresponding relatively low diffusivity of CAT and the relatively large size of this protein molecule compared to the nanoscale pore diameters. As the CAT molecules have a hydrodynamic diameter of approximately 10.2 nm, the pore diameters in the low porosity layers of the PSi sample become substantially reduced in half from 20 ± 5 nm to about 10 ± 5 nm upon capturing one CAT molecule. Electrostatic repulsion between protein molecules and steric hindrance in the pore entrance significantly reduce the probability of CAT protein molecules continuing to enter the pores.

Replacing a closed-ended flow-over PSi microcavity with an open-ended flow-through PSi microcavity membrane should enable more efficient transport of analyte to the in-pore sensing surface based on the presented simulation results. Accordingly, we performed the same molecule adsorption experiments with open-ended PSi microcavity membranes. The sensor response for 3-APTES adsorption was similarly fast in both flow schemes due to non-hindered pore entry for these small molecules and their fast diffusive transport. For larger analytes, there is little benefit to using the flow-through scheme for HRP adsorption since less than a twofold improvement in response time was obtained as compared to the flow-over scheme; however, for CAT adsorption, the flow-through membrane provided a much faster response. The time for the PSi microcavity to reach a 1-nm wavelength shift, a readily measurable value, was one fourth that when the flow-through scheme was utilized. The experimental results indicate that the flow-through PSi membrane is most beneficial for analysis of analytes that can enter the pores but with relatively large dimensions such as antibodies, large proteins, and long nucleic acids. Those molecules, whose diffusivities are on the order of 10^−11^ m^2^/s, also exhibit the greatest improvement ratio by the flow-over format to the flow-through format in their simulated equilibrium times. In contrast, small analytes such as 3-APTES and HRP, whose diffusivities are faster than 10^−10^ m^2^/s, show no significant decreases in response time in both experimental and simulation results when employing the flow-through approach.

## Conclusions

In this work, the analyte transport and equilibrium time of open-ended, flow-through porous membranes were investigated via finite element method simulations and compared to conventional closed-ended, flow-over porous sensors. The simulation results indicate that the flow-through scheme is most beneficial for facilitating the transport of large analytes with slow diffusivity throughout the nanoscale pores using modest flow velocities of 5–10 μL/min. Additionally, the flow-through scheme enables more reasonable response times for the detection of dilute analytes (at <1 μM) and reduces the volume of solution required for analysis. Experimental confirmation of the simulation results was obtained by comparing the performance of closed-ended PSi microcavity sensors in the flow-over scheme and open-ended PSi microcavity membrane sensors in the flow-through scheme. The open-ended PSi microcavity membrane exhibited a fourfold faster response when exposed to the large, 247.5-kDa CAT protein, as the flow-through scheme facilitated improved mass transport. For the adsorption of smaller molecules—3-APTES (221 Da) and HRP (44 kDa)—little to no sensor performance improvement was observed as the closed-ended PSi microcavities did not suffer significant mass transport challenges with these molecules. This work may serve as a guide to determine the benefits of employing a flow-through scheme for porous sensors under given kinetic conditions.

## Abbreviations

3-APTES: 3-Aminopropyltriethoxysilane
CAT: Catalase
DI water: Deionized water
HRP: Horseradish peroxidase
PBS: Phosphate-buffered saline
PDMS: Polydimethylsiloxane
PSi: Porous silicon
SEM: Scanning electron microscope
